# Duration of antibiotic therapy for multidrug resistant *Pseudomonas aeruginosa* pneumonia: is shorter truly better?

**DOI:** 10.1186/s12879-024-09600-w

**Published:** 2024-09-03

**Authors:** Clover N. Truong, Nafeesa Chin-Beckford, Ana Vega, Kailynn DeRonde, Julio Simon, Lilian M. Abbo, Rossana Rosa, Christine A. Vu

**Affiliations:** 1 Department of Pharmacy Services, Jackson Memorial Hospital, Miami, FL USA; 2 Norton Infectious Diseases Institute, Norton Healthcare, 4950 Norton Healthcare Blvd, Suite 303, Louisville, KY 40241 USA; 3 Department of Medicine, Division of Infectious Disease, University of Miami Miller School of Medicine, Miami, FL USA; 4 Department of Infection Prevention and Control, Jackson Health System, Miami, FL USA

**Keywords:** Pneumonia, Treatment duration, *Pseudomonas*, Multidrug resistant

## Abstract

**Background:**

The 2016 IDSA guideline recommends a treatment duration of at least 7 days for hospital-acquired (HAP)/ventilator-associated pneumonia (VAP). The limited literature has demonstrated higher rates of recurrence for non-glucose fermenting gram-negative bacilli with short course therapy, raising the concern of optimal treatment duration for these pathogens. Therefore, we aimed to compare the outcomes for patients receiving shorter therapy treatment (≤ 8 days) versus longer regimen (> 8 days) for the treatment of multidrug resistant (MDR) *Pseudomonas* pneumonia.

**Methods:**

A single-center, retrospective cohort study was conducted to evaluate adult patients receiving an antimicrobial regimen with activity against MDR *Pseudomonas aeruginosa* in respiratory culture between 2017 and 2020 for a minimum of 6 consecutive days. Exclusion criteria were inmates, those with polymicrobial pneumonia, community-acquired pneumonia, and infections requiring prolonged antibiotic therapy.

**Results:**

Of 427 patients with MDR *P. aeruginosa* respiratory isolates, 85 patients were included. Baseline characteristics were similar among groups with a median age of 65.5 years and median APACHE 2 score of 20. Roughly 75% had ventilator-associated pneumonia. Compared to those who received ≤ 8 days of therapy, no difference was seen for clinical success in patients treated for more than 8 days (80% vs. 65.5%, *p* = 0.16). The number of 30-day and 90-day in-hospital mortality, 30-days relapse, and other secondary outcomes did not significantly differ among the treatment groups.

**Conclusions:**

Prolonging treatment duration beyond 8 days did not improve patient outcomes for MDR *P. aeruginosa* HAP/VAP.

## Background

Nosocomial pneumonia is one of the most common hospital-acquired infections [1]. Non-fermenting gram-negative bacterial pathogens – particularly *Pseudomonas aeruginosa* – are common causes of nosocomial pneumonia. *P. aeruginosa* harbor several antimicrobial resistance mechanisms to standard β-lactams; and thus, infections caused by this pathogen are often difficult to treat.

The Centers for Disease Control and Prevention recognizes multidrug resistant (MDR) *P. aeruginosa* as a serious public health threat due to the high burden of mortality and health-care expenditure [2]. Optimal duration of treatment for *P. aeruginosa* HAP/VAP has been a matter of debate to ensure successful outcomes. The 2016 Infectious Diseases Society of America (IDSA) HAP/VAP guidelines recommend treating nosocomial pneumonia for a minimum of 7 days based on clinical improvement [3], but with a mention that recurrence may be increased when treating VAP caused by non-glucose fermenting gram-negative bacilli with a short course of antibiotics (7–8 days) [4, 5]. Previous studies supporting IDSA guideline recommendation do not specifically look at outcomes in patients with pneumonia caused by MDR *P. aeruginosa* and often exclude immunocompromised patient population in whom relapsed pneumonia might lead to a higher mortality beyond 30 days. In these difficult to treat infections in vulnerable patient populations, clinicians may be inclined to treat for a longer duration; however, this may have negative consequences in the future as each additional day of exposure to any anti-pseudomonal antibiotics is associated with an increased risk of new resistance development [6, 7]. Therefore, the purpose of this study is to compare outcomes for patients receiving shorter regimens (≤ 8 days) versus longer regimens (> 8 days) for the treatment of MDR *P. aeruginosa* hospital-acquired/ventilator-associated pneumonia, including those at a higher risk of deaths and/or with immunocompromised conditions.

## Materials and methods

### Study design and patient population

This single-center retrospective cohort study was conducted at Jackson Memorial Hospital in Miami, Florida. The microbiology database queried to identify all patients with respiratory cultures of MDR *P. aeruginosa* isolates from January 2017 through December 2020. MDR *P. aeruginosa* was defined as isolates that were non-susceptible (intermediate or resistant) to one or more drugs in at least three of the following categories: extended spectrum (ES) cephalosporins (i.e. cefepime, ceftazidime), ES penicillin with beta lactamase inhibitor (i.e. piperacillin/tazobactam), fluoroquinolones, aminoglycosides, and/or carbapenems. All adult recipients initiated on systemic antimicrobial regimen active (susceptible in vitro) against MDR *P. aeruginosa* for a minimum of 6 days were eligible for inclusion. Recipients who were inmates, had community acquired pneumonia, polymicrobial pneumonia, empyema, lung abscesses, or other pulmonary complications secondary to pneumonia, non-*P. aeruginosa* infection and/or patients requiring prolonged antibiotic therapy (> 21 days) for a different indication were excluded from the study. This study was approved by the Institutional Review Board of University of Miami-Jackson Health (IRB# 20210568) and a waiver of informed consent was granted.

Inpatient encounter notes along with laboratory records of all eligible patients were retrospectively reviewed. Information extracted included patient demographics, pre-existing comorbidities, Charlson comorbidity index, quick Sepsis-related Organ Failure Assessment (qSOFA) score, Acute Physiology and Chronic Health Evaluation II (APACHE II) score, mechanical ventilation duration prior and after index culture, ICU and hospital length of stays after index culture, vasopressor use within 24 h of index culture, and pneumonia clinical course. Patient specific antibiotic regimen (drug, dose, route of administration, total duration) was also recorded.

### Study outcomes and definitions

The primary outcome was clinical success at end-of-therapy defined as resolution of signs and symptoms of infection and no requirement for additional antibacterial treatment for the same indication. Secondary outcomes included (1) All-cause in-hospital mortality within 30 and 90 days; (2) incidence of relapsed pneumonia within 30 days of index culture defined as reappearance of signs and symptoms of pneumonia with re-isolation of *P. aeruginosa* isolate in the respiratory culture necessitating antibiotic treatment; (3) ICU and hospital length of stay after index culture; (4) mechanical ventilation free-days after index culture, and (5) 30-day readmission rate for pneumonia due to *Pseudomonas aeruginosa*. All outcomes were assessed until discharge or death, or date lost to follow-up.

### Statistical methods

Descriptive statistics were reported using means and standards deviations for normally distributed continuous data, medians and interquartile ranges for non-normally distributed continuous data or ordinal scale data, and percentages for event rates and nominal data. Student’s t-test was used for parametric continuous variables, and Chi-square test, Fisher exact test, or Mann Whitney U was used for categorical variables or non-parametric continuous data as appropriate. Odds ratios for clinical success, 30-day mortality, and 90-day mortality were estimated using logistic regression and adjusted for pre-defined relevant exposure variables (age, intensive care unit stay, vasopressors, mechanical ventilation, qSOFA score, and receiving combination therapy). Statistical analyses were performed with Stata version 14 (College Station, TX).

## Results

### Patient characteristics

From January 2017 through December 2020, 427 MDR *P. aeruginosa* respiratory isolates were identified at Jackson Memorial Hospital. Of these, 85 unique patients met inclusion criteria, 342 patients were excluded due to no active treatment (*n* = 148), polymicrobial pneumonia (*n* = 132), empyema or concomitant infections requiring prolonged antibiotics (*n* = 19), and respiratory cultures obtained within 48 h of admission (*n* = 43). Patients were predominantly male with median age of 63–65 years old. For treatment duration, 30 patients (35.3%) received ≤ 8 days of antibiotics, 55 patients (64.7%) received more than 8 days of antibiotics. The baseline characteristics of the included patients were similar among groups and are summarized in Table 1. The most common indication was ventilator-associated pneumonia among two groups. Median duration of mechanical ventilation prior to index culture was numerically longer in ≤ 8-day group (24.6 days) compared to more than 8 days group (13.6 days). Approximately 15% had history of solid organ transplant. More patients were in the ICU in the more than 8-day group (80%) compared to ≤ 8-day group (63.3%). The median APACHE II score approximated 20 and were not different among treatment groups.

**Table 1:** Baseline characteristics

Baseline Characteristics	≤ 8 days (*n* = 30)	> 8 days (*n* = 55)	*P*-value
Age, median (IQR) years	65.5 (55–70)	63 (42–73)	0.74
Male sex, *n* (%)	22 (73.3)	43 (78.2)	0.62
Ventilated-associated pneumonia, *n* (%)	26 (86.7)	38 (69.1)	0.12
Duration of mechanical ventilation prior to index culture, median (IQR) days	24.6 (11.5–35.4)	13.6 (5.4–28.3)	0.09
Chronic trach prior to index culture, *n* (%)	10 (33.3)	10 (18.2)	0.12
Asthma, *n* (%)	0	1 (1.8)	0.46
COPD, *n* (%)	5 (16.7)	3 (5.4)	0.09
Solid organ transplant, *n* (%)	5 (16.7)	8 (14.5)	0.80
Kidney, *n* (%) Liver, *n* (%) Multivisceral, *n* (%) Lung, *n* (%) Liver-kidney, *n* (%) Kidney-heart, *n* (%)	4 (13.3) 1 (3.3) 0 0 0 0	2 (3.6) 1 (1.8) 1 (1.8) 2 (3.6) 1 (1.8) 1 (1.8)	
Active cancer, *n* (%)	0	6 (10.9)	0.06
HIV, *n* (%)	1 (3.3)	1 (1.8)	0.66
Diabetes, *n* (%)	8 (26.7)	18 (32.7)	0.56
Chronic kidney disease, *n* (%)	7 (23.3)	10 (18.2)	0.57
qSOFA score, median (IQR)	2 (1–2)	2 (2–3)	0.31
APACHE II score, median (IQR)	20.5 (15–28)	20 (17–27)	0.80
Charlson comorbidity index, median (IQR)	4 (2–5)	4 (1–8)	0.43
ICU status at index culture, *n* (%)	19 (63.3)	44 (80)	0.09
Vasopressor within 24 h of index culture, *n* (%)	2 (6.7)	11 (20)	0.10

Definitions: COPD = chronic obstructive pulmonary disease, HIV = human immunodeficiency, ICU = intensive care unit, IQR = interquartile range, vasopressor = norepinephrine, vasopressin, phenylephrine

### Primary and secondary outcomes

At the end-of-treatment, compared to patients who treated with ≤  8 days of antibiotics, there was no significant difference in clinical success for those treated for more than 8 days (OR 0.47 (95% CI 0.16–1.36; *p* = 0.16). Estimates were unchanged after adjusting for age, ICU admission, mechanical ventilation, vasopressors, qSOFA score, and receiving combination therapy (*p* = 0.34). Similarly, there was no difference in 30-day in-hospital mortality among treatment groups [≤ 8-day; 8/30 (26.7%) vs. more than 8 days; 17/55 (30.9%)] (*p* = 0.68). Patients who were given a shorter duration of therapy (≤ 8 days) did not demonstrate a higher relapse rate within 30 days of index culture compared to those who were treated for longer duration of antibiotics (10% vs. 18.2%, *p* = 0.32). Additionally, 90-day in-hospital mortality did not differ among treatment group (*p* = 0.89). As reported in Table 2, none of the other secondary outcome events—number of mechanical ventilation–free days, length of ICU stay, and incidence of 30-day readmission due to *P. aeruginosa* pneumonia – differed significantly among those who were treated ≤ 8 days vs. more than 8 days.

**Table 2:** Primary and secondary outcomes

Proportion of patients experiencing the primary and secondary outcomes of interest			
Outcomes	≤ 8 days (*n* = 30)	> 8 days (*n* = 55)	*P*-value
Clinical success at end-of therapy	24 (80.0)	36 (65.5)	0.16
30-day in-hospital mortality	8 (26.7)	17 (30.9)	0.68
90-day in-hospital mortality	11 (36.7)	21 (38.2)	0.89
30-day re-admission due to pneumonia	0	1	0.99
30-day relapse, *n* (%)	3 (10.0)	10 (18.2)	0.32
Mechanical ventilation-free days, median (IQR)	14 (0–22.5)	16 (5-23.6)	0.70
ICU length of stay, median (IQR)	17 (8–30)	17 (12–32)	0.90
**Unadjusted and adjusted odds ratios for the effect of treatment duration on clinical success**, **30-day and 90-day mortality**			
Unadjusted odds ratio of clinical success	Baseline	0.47 (0.16–1.36)	0.16
Adjusted odds ratio of clinical success*	Baseline	0.58 (0.19–1.80)	0.34
Unadjusted odds ratio of 30-day mortality	Baseline	1.23 (0.46–3.31)	0.68
Adjusted odds ratio of 30-day mortality*	Baseline	0.93 (0.28–3.06)	0.91
Unadjusted odds ratio of 90-day mortality	Baseline	1.07 (0.42–2.68)	0.89
Adjusted odds ratio of 90-day mortality*	Baseline	0.70 (0.23–2.08)	0.52

Definitions: ICU = intensive care unit, IQR = interquartile range *Model adjusted for: age, ICU, vasopressors, mechanical ventilation, qSOFA score, receiving combination therapy

### Antibiotic regimen

Since antibiotic regimens were chosen at the discretion of treating physicians and based on susceptibility reports, various antibiotics were used during the study period (Table 3).

**Table 3:** Antibiotic regimens

Antibiotic Regimen	≤ 8 days (*n* = 30)	> 8 days (*n* = 55)	*P*-value
Monotherapy, *n* (%) Meropenem Cefepime Ceftolozane-tazobactam Cetazidime Ceftazidime-avibactam Piperacillin-tazobactam Levofloxacin IV/PO Ciprofloxacin IV Colistin IV	17 (56.7%) 5 8 3 1 1 1 0 0 0	32 (58.2%) 2 14 5 4 0 3 4 1 1	0.89
Combination therapy, *n* (%) Ceftolozane-tazobactam/ceftazidime-avibactam + aztreonam Ceftolozane-tazobactam + tobramycin inhalation Ceftolozane-tazobactam + tobramycin IV Ceftolozane-tazobactam + polymyxin B IV/amikacin IV Colistin IV/ceftolozane-tazobactam + tobramycin inhalation Ceftolozane-tozobactam + levofloxacin Meropenem + amikacin IV Meropenem + colistin IV Cefepime + tobramycin inhalation Cefepime + tobramycin IV Cefepime + amikacin inhalation Cefepime + gentamicin IV Ceftazidime-avibactam + aztreonam Ceftazidime-avibactam/ceftolozane-tazobactam + polymyxin B IV Ceftazidime-avibactam + amikacin IV Levofloxaxin + tobramycin inhalation Polymyxin B IV + colistin inhalation Ceftolozane-tazobactam + tobramycin Ceftazidime + colistin inhalation Ciprofloxacin IV + tobramycin inhalation Levofloxacin + amikacin IV Colistin IV + levofloxacin Meropenem + tobramycin IV	13 (43.3%) 1 4 0 0 1 0 1 1 1 0 0 0 1 0 0 1 1 1 0 0 0 0 0	23 (41.8%) 0 6 1 1 0 1 0 0 3 1 1 1 1 1 1 0 0 0 1 1 1 1 1	

## Discussion

Multidrug resistant *P. aeruginosa* continues to pose a threat to hospitalized patients, especially those with immunocompromised conditions. To our knowledge, this is the first study assessing the outcomes of shorter course vs. longer course of antibiotics specifically in patients with HAP/VAP caused by MDR *Pseudomonas aeruginosa*. We found longer courses of antibiotics (> 8 days) did not result in any significant difference in outcomes compared to those treated with shorter therapy. This is contrary to a recent randomized controlled trial conducted by Bougle et al. where non-inferiority of short duration (8 days) in the treatment of *P. aeruginosa* VAP was not demonstrated compared to long duration (15 days) due to significant higher rate of recurrence in the shorter duration group [8], however this study was limited due to its lack of power. Our clinical success at the end of therapy in patients treated with 8 days of antibiotics (80%) was very similar to that of the REPROVE trial where 79.5% of their patients with *P. aeruginosa* VAP were successfully treated with ceftazidime-avibactam for 7–14 days [9]. Furthermore, all-cause mortality in our cohort (26.7%) was similar to patients being treated with 14 days of imipenem-relebactam for *P. aeruginosa* HAP/VAP in the RESTORE-IMI 2 trial (33.3%) [10]. Lastly, for the ASPECT-NP trial (50/511; 9.78% patients with MDR *Pseudomonas aeruginosa)*, median duration of treatment was 12 days (range 0–14) with clinical cure at test-of-cure (7–14 days after end-of-therapy) was 60% and 28-day all-cause mortality was 16.7% in ceftolozane-tazobactam vs. 26.3% in meropenem group [11]. One of the biggest strengths of our study was the population consisting mostly of patients at increased risk of adverse treatment outcomes and death. Majority of our cohort were in the ICU at time of infection (> 60%), had a median APACHE II score of 20, and we included a subset of immunocompromised patients. Despite the high acuity of our patient population and being treated for an MDR infection, we still did not find worse outcome with shorter course of antibiotics. This result favors the approach of treating patients with MDR *P. aeruginosa* HAP/VAP for ≤8 days, instead of a longer duration, to prevent the development of resistance and adverse drug events.

The use of combination therapy for severe pseudomonal infections has been considered standard of practice by many clinicians due to in-vitro antibiotic synergy and potential prevention of resistance emergence while receiving therapy. In our cohort, the choice of antibiotics for treatment of MDR *P. aeruginosa* HAP/VAP was dictated by the susceptibility patterns, with the majority using monotherapy. We did not see any trend favoring combination therapy over monotherapy. This finding was similar to the meta-analysis conducted by the IDSA expert panels including 7 randomized trials which found that combination therapy offered no benefit in reducing mortality beyond monotherapy (RR, 0.94; 95% CI, 0.76–1.16) [3]. In a retrospective cohort study that included 183 episodes of VAP caused by *Pseudomonas aeruginosa*, Garnacho-Montero et al. found that inappropriate empiric therapy was associated with increased mortality. After exclusion of patients receiving inappropriate empiric treatment regimen, mortality was not different among groups who were treated with monotherapy vs. combination (23.1% vs. 33.2%, adjusted HR 0.9; 95% CI 0.5–1.63) [12]. This observational study along with the meta-analysis by IDSA panel suggested that once the antibiotic susceptibilities were known, combination therapy was not necessary for *P. aeruginosa* HAP/VAP.

Limitations of the present study should be noted. Our sample sizes were small; and thus, may not be powered enough to detect the difference in outcomes among treatment groups. However, outcomes including clinical success rate at end of therapy and in-hospital mortality in our cohort were similar to those reported in previous trials with no trend favoring longer course of treatment. Additionally, since this was a retrospective study, clinical diagnosis of pneumonia was largely dependent on provider documentation, which did not always detail the specific rationale for the diagnosis and thus it was hard to retrospectively differentiate between pneumonia and possible colonization. Finally, we were unable to capture mortality or re-admission events outside our hospital; and thus, the incident rate may represent an underestimation.

## Conclusions

MDR *P. aeruginosa* remains a significant pathogen in nosocomial pneumonia and is associated with high mortality. In this study, treating MDR *P. aeruginosa* HAP/VAP beyond 8 days did not result in better clinical success, lower mortality, or less incidence of relapse. Therefore, shorter course of antibiotics (≤8 days) can be considered in treating MDR *P. aeruginosa* HAP/VAP, including those with immunocompromised conditions. Further studies are needed to validate our initial findings.

## Data Availability

The datasets used in this study are available from the corresponding author on reasonable request.

## Abbreviations

APACHE II: Acute Physiology and Chronic Health Evaluation II (severity of disease classification system utilizing initial values of 12 physiologic measurements, age, and previous health status)
CCI: Charlson Comorbidity Index (a weighted score taking into account the number and severity of comorbidity conditions)
HAP: Hospital-acquired pneumonia
IDSA: Infectious Diseases Society of America
MDR: Multidrug resistant
qSOFA: Quick Sepsis-related Organ Failure Assessment (bedside clinical tool to identify high risk patients with suspected infections)
VAP: Ventilator-associated pneumonia
